# Supplementary material for: MSC-Derived Exosomes Protect Vertebral Endplate Chondrocytes against Apoptosis and Calcification via the miR-31-5p/ATF6 Axis
Source: Mol Ther Nucleic Acids. 2020 Sep 26;22:601–14. doi: 10.1016/j.omtn.2020.09.026 (PMC7569190; doi:10.1016/j.omtn.2020.09.026)
Supplement: Document S1. Figures S1 and S2 and Tables S1 and S2 [file mmc1.pdf]

OMTN, Volume *22*

Supplemental Information

MSC-Derived Exosomes Protect Vertebral Endplate Chondrocytes against Apoptosis and Calcification via the miR-31-5p/ATF6 Axis

Lin Xie, Zhenhao Chen, Ming Liu, Weibo Huang, Fei Zou, Xiaosheng Ma, Jie Tao, Jingkang Guo, Xinlei Xia, Feizhou Lyu, Hongli Wang, Chaojun Zheng, and Jianyuan Jiang

Figure S1

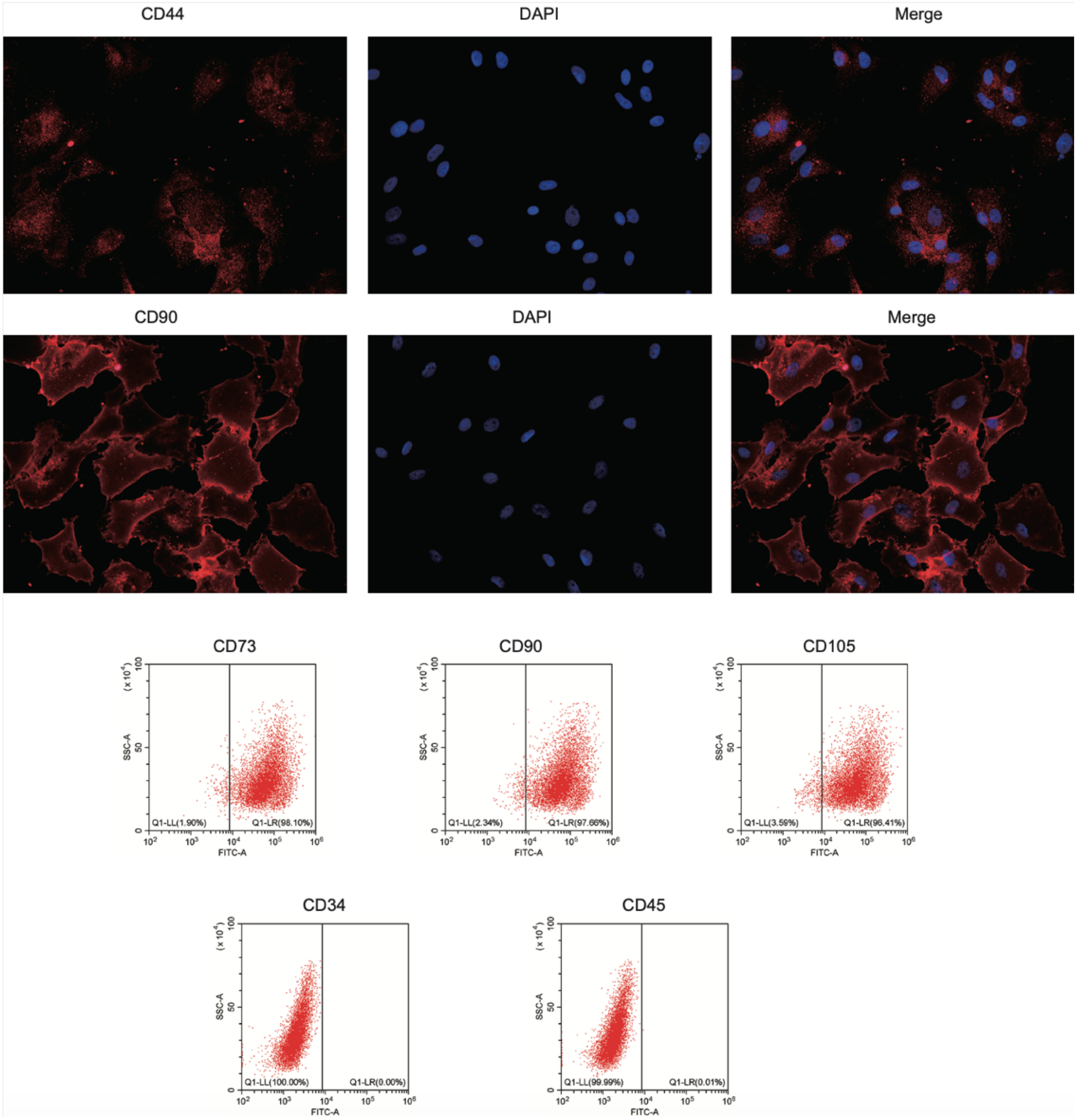

Figure S1. Analysis of expression patterns of CD44, CD105, CD73, and CD90, and deficiency of CD45 and CD34 surface molecules on MSCs using Immunofluorescence or flow cytometry.

Figure S2

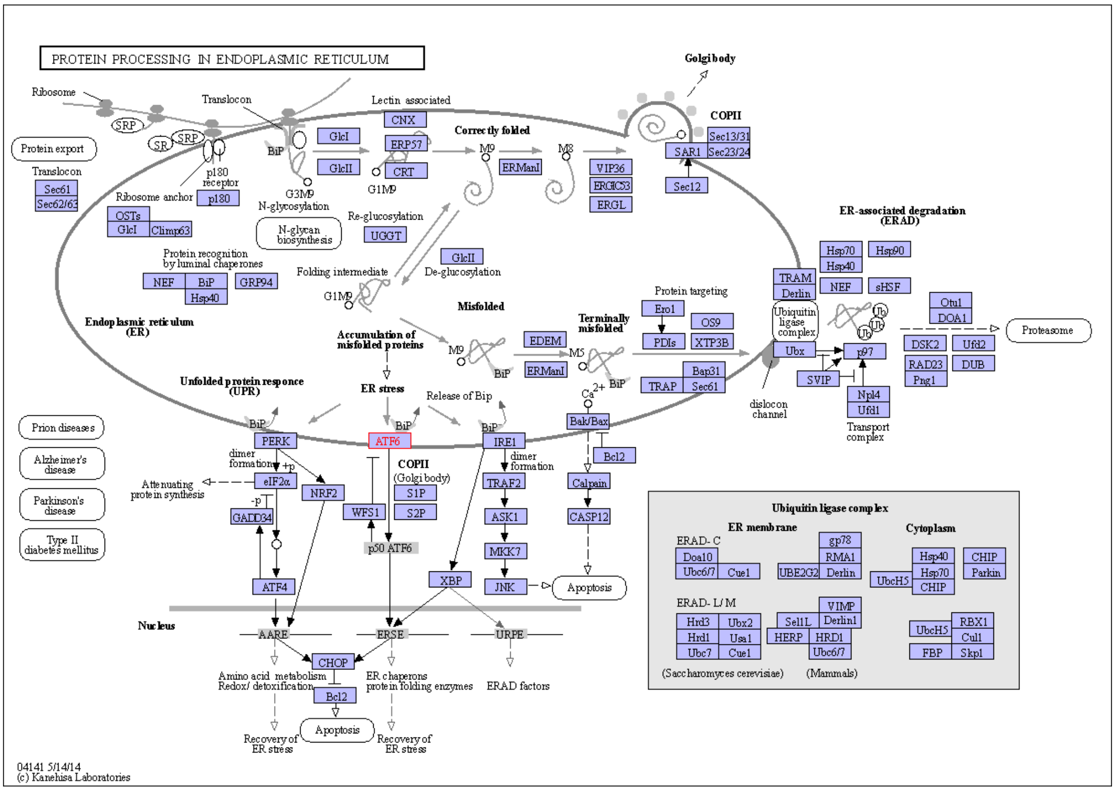

Figure S2. KEGG pathway showed that the direct effect of ATF6 was ER stress activation and related apoptosis pathway.

Table S1. MSCs and Endplate donors used for this study.

Donor ID	Sex	Age	Surgery Site	Site of vertebral body for MSCs	Site of intervertebral disc for EPCs
#1	Female	19	C4/5, C5/6		C5/6
#2	Male	27	C4/5, C5/6, C6/7		C5/6
#3	Male	25	C4/5, C5/6, C6/7		C5/6
#4	Male	28	C4/5, C5/6		C4/5
#5	Male	20	C4/5, C5/6, C6/7		C4/5
#6	Male	23	C4/5, C5/6		C4/5
#7	Male	21	C4/5, C5/6		C4/5
#8	Male	21	C4/5, C5/6, C6/7		C4/5
#9	Female	45	C4/5, C5, C5/6	C5	C4/5
#10	Female	53	C4/5, C5,	C5	C5/6

			C5/6		
#11	Male	49	C3/4, C4, C4/5	C4	C4/5
#12	Female	48	C4/5, C5/6		C4/5
#13	Male	49	C4/5, C5, C5/6	C5	C4/5
#14	Male	47	C3/4, C4, C4/5	C4	C4/5
#15	Female	47	C4/5, C5/6		C4/5
#16	Female	59	C4/5, C5/6		C4/5

MSCs, mesenchymal stem cells; EPCs, Endplate Chondrocytes; C, cervical vertebral body;

Table S2. Sequence used in this study.

	Sequences
U6 Forward	5'-CTCGCTTCGGCAGCACA-3'
U6 Reversed	5'-ACGCTTCACGAATTTGCGT-3'
miR-31-5p Forward	5'-CGGCGGAGGCAAGATGCTGGCA-3'
miR-31-5p Reversed	5'-CAACTGGTGTCGTGGAGTCGG-3'
agomiR-31-5p	AGGCAAGAUGCUGGCAUAGCU
agomiR-NC	UUUGUACUACACAAAAGUACU
antagomiR-31-5p	CAGCUAUGCCAGCAUCUUGCCU
Antagomir-NC	AAACAUGAUGUGUUUUCAUGAC
ATF6-siRNA Sense	AGTCGCCTTTTAGTCCGGTTC
ATF6-siRNA Antisense	CTGACTCCCAAGGCATCAAAT
Scramble siRNA Sense	ACCACAGTCCATGCCATCAC
Scramble siRNA Antisense	TCCACCACCCTGTTGCTGTA
