# Supplementary material for: MSC-Derived Exosomes Protect Vertebral Endplate Chondrocytes against Apoptosis and Calcification via the miR-31-5p/ATF6 Axis
Source: Mol Ther Nucleic Acids. 2020 Sep 26;22:601–14. doi: 10.1016/j.omtn.2020.09.026 (PMC7569190; doi:10.1016/j.omtn.2020.09.026)
Supplement: Document S2. Article plus Supplemental Information [file mmc2.pdf]

Molecular Therapy
Nucleic Acids
Original Article

MSC-Derived Exosomes Protect Vertebral Endplate Chondrocytes against Apoptosis and Calcification via the miR-31-5p/ATF6 Axis

Lin Xie,[1,5] Zhenhao Chen,[1,5] Ming Liu,[2,5] Weibo Huang,[1] Fei Zou,[1] Xiaosheng Ma,[1] Jie Tao,[3] Jingkang Guo,[3] Xinlei Xia,[1] Feizhou Lyu,[1,4] Hongli Wang,[1] Chaojun Zheng,[1] and Jianyuan Jiang[1]

[1]Department of Orthopedics, Huashan Hospital, Fudan University, Shanghai 200040, China; [2]Department of Orthopedic Surgery, Wuhan Fourth Hospital, Huazhong University of Science and Technology, Wuhan 430000, China; [3]Laboratory of Neuropharmacology and Neurotoxicology, Shanghai University, Shanghai 200436, China; [4]Department of Orthopedic Surgery, The Fifth People's Hospital of Shanghai, Fudan University, Shanghai 201100, China

Apoptosis and calcification of endplate chondrocytes (EPCs) can exacerbate intervertebral disc degeneration (IVDD). Mesenchymal stem cell-derived exosomes (MSC-exosomes) are reported to have the therapeutic potential in IVDD. However, the effects and related mechanisms of MSC-exosomes on EPCs are still unclear. We aimed to investigate the role of MSC-exosomes on EPCs with a tert-butyl hydroperoxide (TBHP)-induced oxidative stress cell model and IVDD rat model. First, our study revealed that TBHP could result in apoptosis and calcification of EPCs, and MSC-exosomes could inhibit the detrimental effects. We also found that these protective effects were inhibited after miroRNA (miR)-31-5p levels were downregulated in MSC-exosomes. The target relationship between miR-31-5p and ATF6 was tested. miR-31-5p negatively regulated ATF6-related endoplasmic reticulum (ER) stress and inhibited apoptosis and calcification in EPCs. Our *in vivo* experiments indicated that sub-endplate injection of MSC-exosomes can ameliorate IVDD; however, after miR-31-5p levels were downregulated in MSC-exosomes, these protective effects were inhibited. In conclusion, MSC-exosomes reduced apoptosis and calcification in EPCs, and the underlying mechanism may be related to miR-31-5p/ATF6/ER stress pathway regulation.

INTRODUCTION

Intervertebral disc (IVD) degeneration (IVDD) leads to a series of spinal degenerative disc diseases that resulted in a huge burden on the global healthcare system.[1,2] The IVD is composed of the out-surrounded annulus fibrosus (AF), inner nucleus pulposus (NP), and up-down cartilaginous endplate (CEP). IVD is the largest avascular structure in the human body and receives all nutrients from the bone marrow of adjacent vertebral bodies.[3] The endplate, CEP and bony endplate (BEP) included, is the nutrition channel. Degeneration of the CEP can act as a significant barrier to nutrient transport in the endplate.[3] Excessive endplate chondrocyte (EPC) apoptosis and calcification are the two major processes of CEP degeneration.[4] In addition, previous studies showed that stem cells in the adjacent vertebral body could migrate to the NP physiologically through the nutrition channel to maintain the IVD environment balance.[5,6] In IVDD, CEP degeneration leads to blockage of the nutrition channel in the endplate, which results in degeneration of the NP and endogenous repair failure.[7,8] Therefore, finding an effective method to alleviate CEP degeneration to prevent or reverse IVDD is necessary.

Exosomes are nanoscale extracellular membrane vesicles (50–150 nm in diameter).[9] When endoplasmic multivesicular bodies (MVBs) are fused with cell membranes, exosomes carrying biomolecules, such as lipids, proteins, and microRNA (miRNA), are released into the extracellular environment.[10] The lipid membrane facilitates the uptake of exosomes by nearby or distant receptor cells. The ingested exosomes have biological activities, including immunomodulation, angiogenesis, autophagy, and stem cell differentiation.[11] Almost all types of cells can produce exosomes.[12] The RNA cargo in exosomes has attracted attention, especially miRNAs.[11] In addition, the lipid membrane of the exosome protects the internal miRNAs from digestion by RNA enzymes.[13]

miRNAs are short noncoding RNAs that modulate numerous biological processes.[14] They interact directly with the complementary sites of the 3′ UTR of the target mRNAs, hence, modulating the degree of degradation.[15] Besides functioning within cells, miRNAs are produced in exosomes and then translocated to proximate or distant cells to modulate gene expression and regulate cell function.[16,17]

Received 16 June 2020; accepted 21 September 2020;
https://doi.org/10.1016/j.omtn.2020.09.026.

[5]These authors contributed equally to this work.

Correspondence: Hongli Wang, Department of Orthopedics, Huashan Hospital, Fudan University, 12 Mid-Wulumuqi Road, Shanghai 200040, China.
E-mail: hlw1234spine@126.com
Correspondence: Chaojun Zheng, Department of Orthopedics, Huashan Hospital, Fudan University, 12 Mid-Wulumuqi Road, Shanghai 200040, China.
E-mail: countd2388@163.com
Correspondence: Jianyuan Jiang, Department of Orthopedics, Huashan Hospital, Fudan University, 12 Mid-Wulumuqi Road, Shanghai 200040, China.
E-mail: 18111220066@fudan.edu.cn

This is an open access article under the CC BY license (http://creativecommons.org/licenses/by/4.0/).

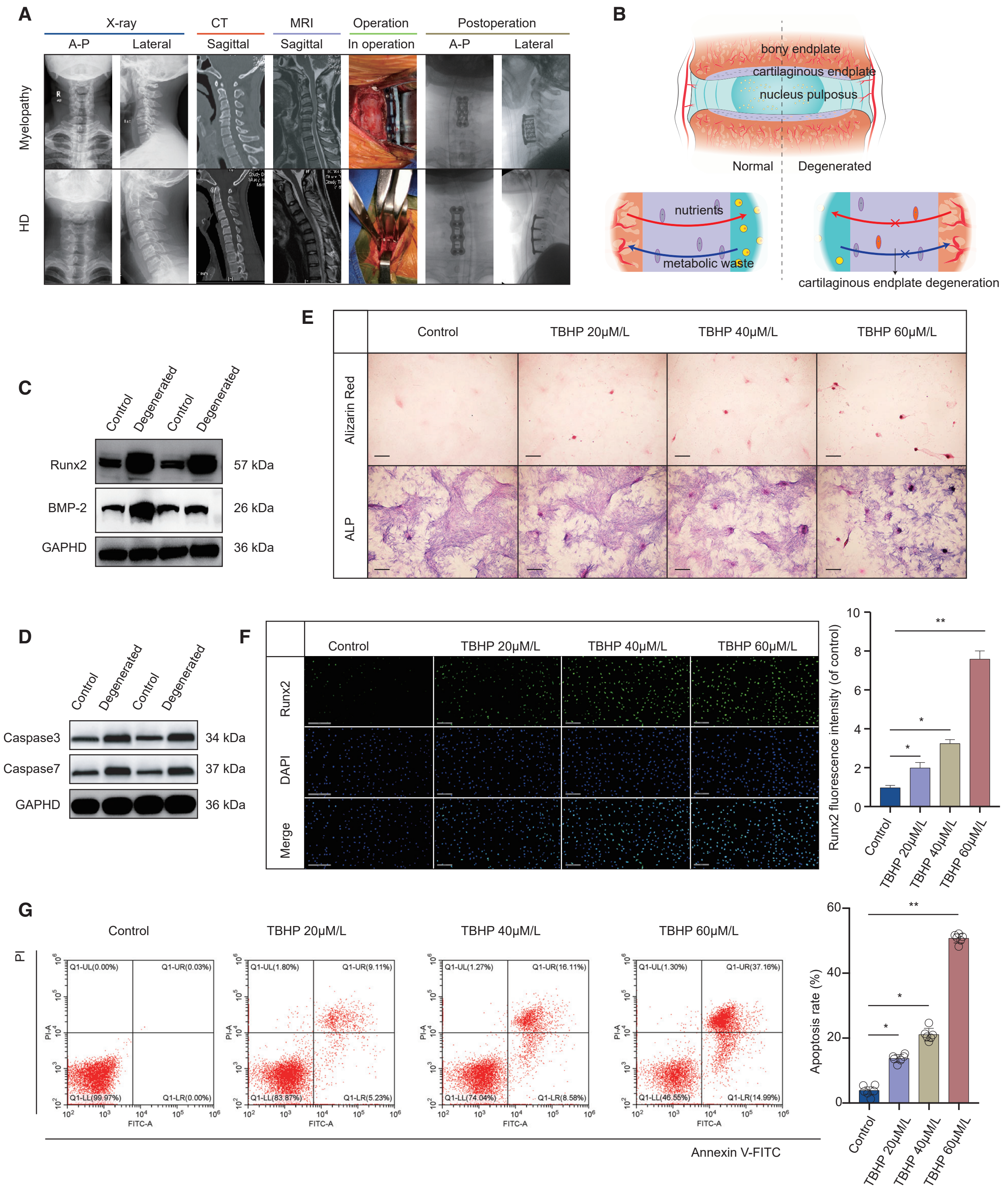

Figure 1. Apoptosis and Calcification in Cartilaginous Endplate (CEP) of IVDD Patients and TBHP-Treated EPCs

(A) Representative plain radiographs, computed tomography (CT), and MRI images of patients with cervical myelopathy or Hirayama disease (HD). (B) Endplate forms a continuing boundary superior and inferior to the intervertebral disc that segregates the vertebra from the inner nucleus pulposus (NP). (C) Runx2 and BMP-2 protein levels were upregulated in CEP from patients with IVDD. (D) Caspase-3 and caspase-7 protein levels were upregulated in CEP from patients with IVDD. (E) Calcification was

(legend continued on next page)

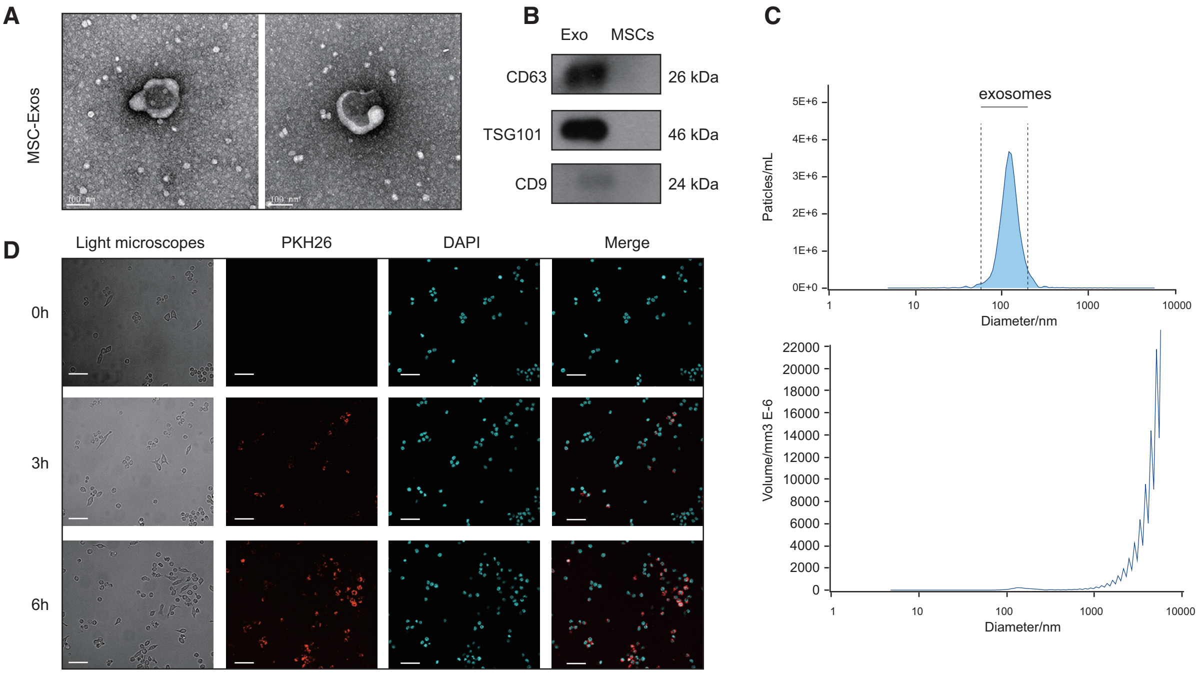

Figure 2. Identification of MSC-Exosomes and MSC-Exosome Uptake by EPCs

(A) Transmission electron micrograph (TEM) of the exosomes secreted from the MSC (scale bars, 100 nm). (B) Immunophenotype of MSC-exosomes. (C) Nano-sight analysis for the particle size. (D) Confocal scanning laser microscopy exhibiting PKH26-labeled exosomes internalized by EPCs for 0, 3, and 6 h (scale bars, 200 μm).

MicroRNA (miR)-31-5p is a commonly downmodulated miRNA in IVDD tissues.[18] In addition, it has been reported that miR-31-5p serves as a negative mediator of calcification and apoptosis.[19] Therefore, we sought to inspect the miR-31-5p expression in mesenchymal stem cell (MSC)-exosomes and their effects in EPCs.

In the current study, we investigated the effects of MSC-exosomes on apoptosis and calcification in EPCs under oxidative stress induced by tert-butyl hydroperoxide (TBHP) and then assessed their effects via sub-endplate injection in a rat-tail IVDD model. We further elucidated the possible mechanism of MSC-exosomes in influencing EPCs.

RESULTS

Apoptosis and Calcification of CEP in IVDD and TBHP Induce Apoptosis and Calcification in EPCs

To investigate apoptosis and calcification of CEP in IVDD, we inspected the expression levels of correlated proteins in 8 pairs of patients with degenerative cervical disc disease as IVDD and Hirayama disease (HD) as control (Figures 1A and 1B; Table S1). The western blot results showed increased expression of apoptosis-related proteins (caspase-3 and caspase-7) and calcification-related proteins (Runx2 and BMP-2) in the CEP of IVDD (Figures 1C and 1D). To explore the calcification of EPCs caused by TBHP, EPCs were treated with different levels of TBHP and then stained with alizarin red and alkaline phosphatase (ALP). Consequently, the calcification of EPCs was increased after TBHP treatment (Figure 1E). Further immunofluorescence staining showed that TBHP treatment distinctly increased the level of Runx2 in EPCs (Figure 1F). Then, flow cytometry was used to detect the impact of distinct concentrations of TBHP on the apoptosis of EPCs. The results showed that the apoptosis of EPCs increased under TBHP treatment (Figure 1G). These results indicated that apoptosis and calcification of CEP in IVDD and oxidative stress (TBHP) induced apoptosis and calcification in EPCs.

Identification of MSC-Exosomes and MSC-Exosome Uptake by EPCs

We used transmission electron microscopy (TEM), dynamic light scattering (DLS), and western blotting to evaluate the exosomes isolated from MSCs. TEM showed that these particles possess a cup-shaped or spherical morphology (Figure 2A), consistent with previous studies.[20,21] Western blotting further validated that these particles possessed exosomal surface markers, including TSG101, CD9, and CD63 (Figure 2B). DLS indicated that the particle size was between 30 and 200 nm (Figure 2C), consistent with previous

upregulated in TBHP-treated EPCs using the alizarin red staining and ALP staining methods (scale bars, 50 μm). (F) Runx2 expression was detected by immunofluorescence analysis (scale bars, 200 μm). (G) Impact of TBHP on EPC apoptosis was examined using flow cytometry assay. Data are mean ± SD. *$p < 0.05$, **$p < 0.01$, ***$p < 0.001$.

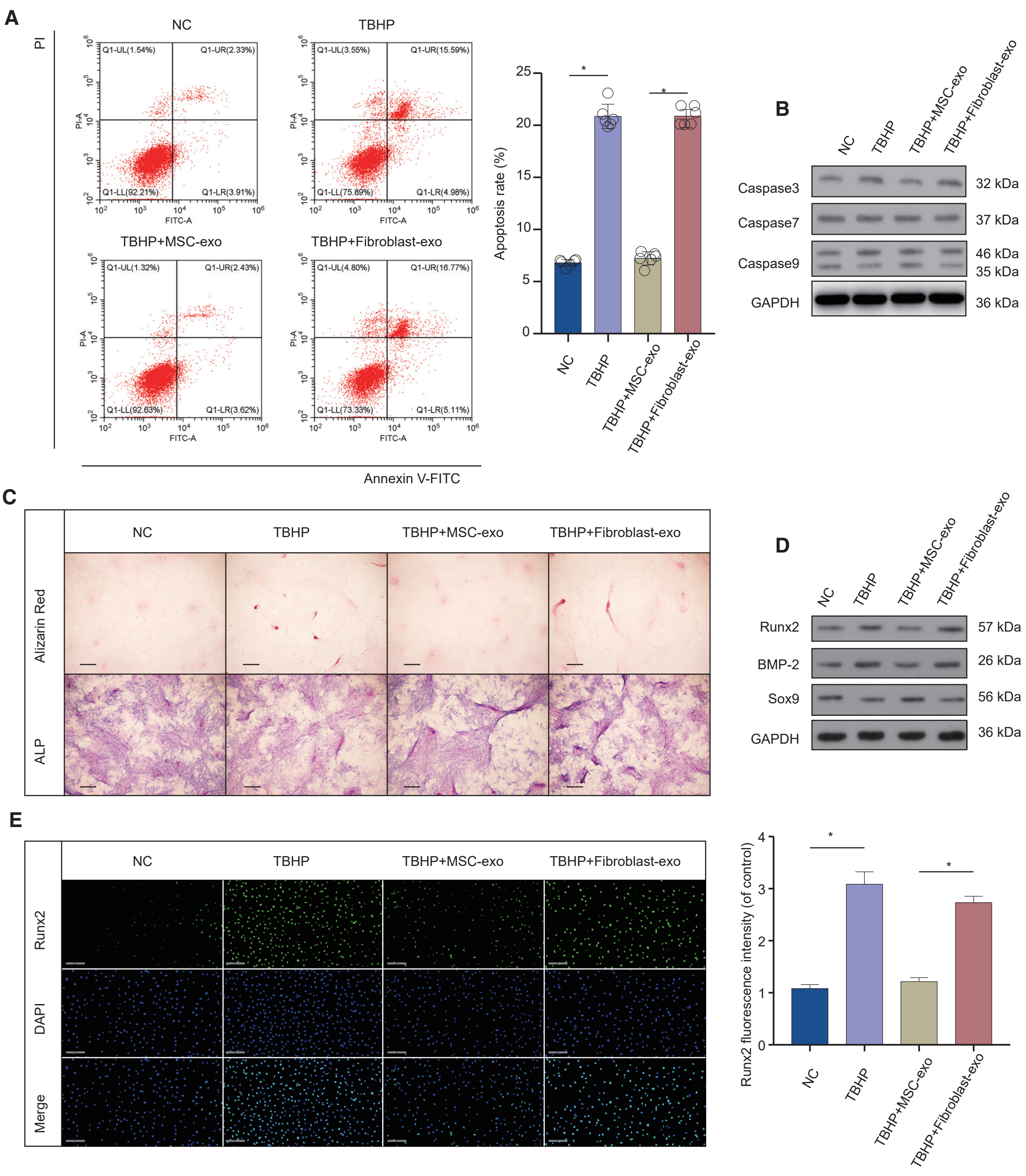

Figure 3. MSC-Exosomes Inhibited Apoptosis and Calcification in EPCs

(A) Anti-apoptosis of MSC-exosomes in TBHP-treated EPCs. (B) Capase-3, caspase-7, and caspase-9 in EPCs after treatment of MSC-exosomes. (C) Alizarin red staining and ALP staining of EPCs after treatment of MSC-exosomes (scale bars, 50 μm). (D) Runx2, BMP-2, and Sox9 in EPCs after treatment of MSC-exosomes. (E) Immunofluorescence of Runx2 protein in EPCs after treatment of MSC-exosomes (scale bars, 200 μm). Data are mean ± SD. *$p < 0.05$, **$p < 0.01$, ***$p < 0.001$.

findings. These results together confirmed that the separated nanoparticles were exosomes. After incubation with EPCs, PKH26-labeled exosomes exhibited red fluorescence in the cytoplasm of EPCs (Figure 2D), implying the MSC-exosome uptake by EPCs.

MSC-Exosomes Inhibited Apoptosis and Calcification in EPCs under Oxidative Stress

Next, we explore the effects of MSC-exosomes on apoptosis and calcification in EPCs under oxidative stress. The MSC-exosomes reduced

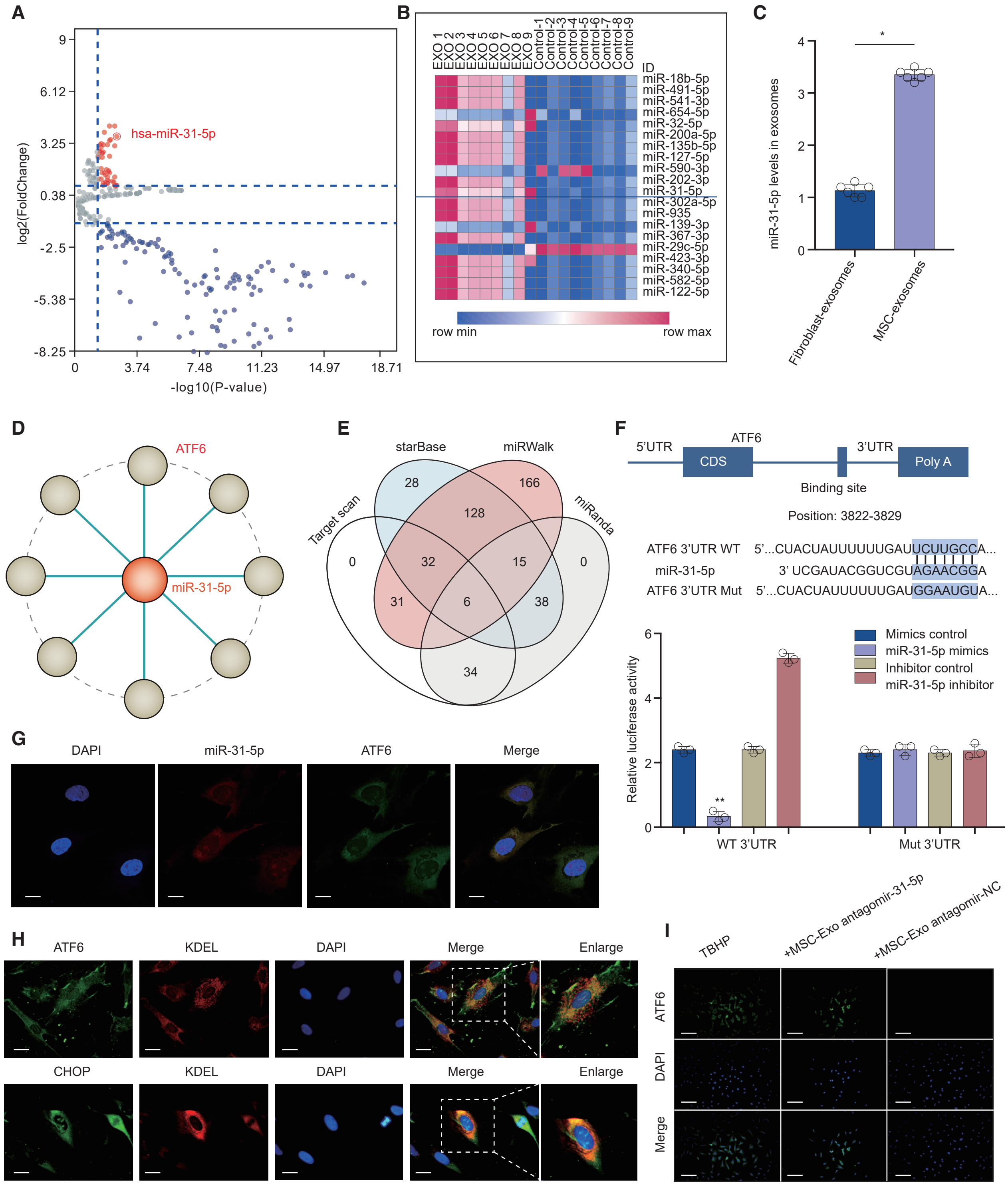

Figure 4. miR-31-5p Was Highly Expressed in MSC-Exosomes, and ATF6 Was a Target of miR-31-5p

(A) Volcano plots depicted differential expression of miRNAs examined using miRNA microarray in MSC-exosomes compared to cells. (B) Heatmap of 20 upregulated miRNAs in microarray. (C) qRT-PCR assay verified the upregulation of miR-31-5p in MSC-exosomes compared to fibroblast-exosomes. (D) Cystoscope was utilized to verify the targets of miR-31-5p. (E) Venn diagram indicated the targets using different algorithms. (F) EPCs were inserted with miR-31-5p and luciferase constructs of ATF6 with the wild-type-putative miR-31-5p binding sites or mutated sites via transfection. (G) FISH revealed that both miR-31-5p and ATF6 mRNA were localized in the cytoplasm. Blue

(legend continued on next page)

the percentage of TBHP-induced apoptosis of EPCs but not fibroblast-exosomes (Figure 3A). At the same time, after treatment of EPCs with MSC-exosomes, the expression levels of activated caspase-3, caspase-7, and caspase-9 decreased but not fibroblast-exosomes (Figure 3B). Alizarin red and ALP staining results revealed that TBHP-induced oxidative stress increased EPC calcification. MSC-exosomes partially inhibited EPC calcification but not fibroblast-exosomes (Figure 3C). Moreover, the western blot results indicated that calcification increased after TBHP treatment, but this impact was reversed by MSC-exosome treatment (Figure 3D). Further immunofluorescence analysis revealed that TBHP treatment significantly increased the relative level of Runx2 in EPCs, whereas MSC-exosome treatment reverses this effect (Figure 3E). These results indicated that MSC-exosomes have protective effects on apoptosis and calcification in EPCs under oxidative stress.

miR-31-5p Was Highly Expressed in MSC-Exosomes, and ATF6 Was the Target of miR-31-5p

We used the microarray to compare the miRNA levels in exosomes and cells. A total of 3,156 capture probes were detected (Figure 4A), and 20 miRNAs were significantly upregulated in MSC-exosomes (Figure 4B). In addition, we found that the content of miR-31-5p in MSC-exosomes was markedly higher compared to the fibroblast-exosomes (Figure 4C). To investigate whether miR-31-5p targets ATF6 directly, we constructed the miRNA-mRNA network using Cytoscape software (https://cytoscape.org/) (Figure 4D). We searched for prospective targets of miR-31-5p and compiled all of the predicted genes for Venn analysis (Figure 4E). Furthermore, the binding regions between miR-31-5p and ATF6 were assessed using the dual-luciferase activity experiment (Figure 4F). We also used the subcellular miRNA and mRNA localization to elucidate the mode of action. The fluorescent *in situ* hybridization (FISH) results revealed that miR-31-5p and ATF6 are both located in the cytoplasm (Figure 4G). Double staining of ATF6, CHOP, and KDEL (endoplasmic reticulum [ER] marker) indicated the action mode in EPCs in the ER (Figure 4H). Furthermore, MSC-exosomes decreased the expression of ATF6 in EPCs, whereas this effect was suppressed by antagomir-31-5p (Figure 4I). These data collectively elucidated that ATF6 was the target of miR-31-5p, and miR-31-5p negatively regulated the ATF6-related ER-stress pathway.

miR-31-5p in MSC-Exosomes Inhibited Apoptosis and Calcification in EPCs under Oxidative Stress

We studied the role of miR-31-5p on TBHP-induced apoptosis and calcification in EPCs by over- and underexpressing miR-31-5p. The agomir-31-5p significantly reduced apoptosis, whereas antagomir-31-5p aggravated apoptosis (Figures 5A and 5B). When the exosomes extracted from the supernatant of MSCs were inserted with antagomir-31-5p via transfection, the exosomes lost their anti-apoptotic effect (Figures 5E and 5F). These results implied that miR-31-5p mediated some therapeutic advantages of MSC-exosomes. Additionally, we explored the effect of the miR-31-5p expression on EPC calcification. Agomir-31-5p remarkably reduced calcification, whereas antagomir-31-5p distinctly aggravated calcification (Figures 5C and 5D). When exosomes were extracted from the supernatant of MSCs inserted with antagomir-31-5p via transfection, the exosomes lost their anti-calcification influence (Figures 5G and 5H). These results collectively indicated that miR-31-5p suppresses apoptosis and calcification in EPCs, and MSC-exosomes inhibits apoptosis and calcification in EPCs via miR-31-5p.

miR-31-5p Exerts Effects in EPCs by Targeting the ATF6-Related ER-Stress Pathway

To elucidate the target genes, as well as molecular cascades of EPCs, pathway analysis indicated the direct role of ATF6 in regulation of apoptosis and calcification via the ER-stress pathway (Figures 6A and S2). After TBHP treatment, the levels of ATF6, apoptosis, and calcification-related proteins in EPCs were significantly increased (Figure 6B). Immunofluorescence staining showed that caspase-12 in EPCs was increased after TBHP treatment (Figure 6C). We incubated the EPCs with miR-31-5p-deficient MSC-exosomes. The suppressing impacts of MSC-exosomes on ER-stress-related apoptosis and calcification were inhibited, implying that MSC-exosomes inhibited the ER-stress-related apoptosis and calcification in EPCs via miR-31-5p (Figure 6D). Signal transduction pathways and expressions of related genes in the GEO database revealed that ATF6-related apoptosis and calcification were increased in IVDD (Figure 6E). Flow cytometry was used to examine the relationship between ATF6 and apoptosis. Compared with the control group, ATF6 small interfering RNA (siRNA) markedly reduced the rate of apoptosis (Figure 6F). These results collectively confirmed that miR-31-5p in MSC-exosomes alleviated apoptosis and calcification in EPCs by targeting the ATF6-related ER-stress pathway.

Sub-Endplate Injection of MSC-Exosomes Ameliorated IVDD in Rat Models

We established the rat IVDD model successfully. Once a week, up to 9 weeks, when they were sacrificed, we injected MSC-exosome sub-endplate in the rat tail. 9 weeks following the injection, the MRI score of the MSC-exosome group was distinctly lower compared to the noninjection group (Figures 7A and 7B). At 9 weeks, histological analysis was performed using hematoxylin and eosin (H&E) staining, Safranin-O staining and Alcian blue staining. H&E staining results showed that the structure of the CEP was confused, the volume of the NP tissue was markedly decreased in the IVDD group, and both CEP and NP tissues in the MSC-exosome group were better preserved (Figure 7C). Safranin-O stains proteoglycans and glycosaminoglycans (red). We found that the CEP was thicker and that the

fluorescence designates the nucleus, red fluorescence designates miR-31-5p, and green fluorescence designates ATF6 mRNA (scale bars, 10 μm). (H) Immunofluorescence double staining for colocalization of ATF6 and CHOP with KDEL were localized in the endoplasmic reticulum (ER) of EPCs (scale bars, 20 μm). (I) Immunofluorescence of ATF6 protein in EPCs (scale bars, 100 μm). Data are mean ± SD. $^{*}p < 0.05$, $^{**}p < 0.01$, $^{***}p < 0.001$.

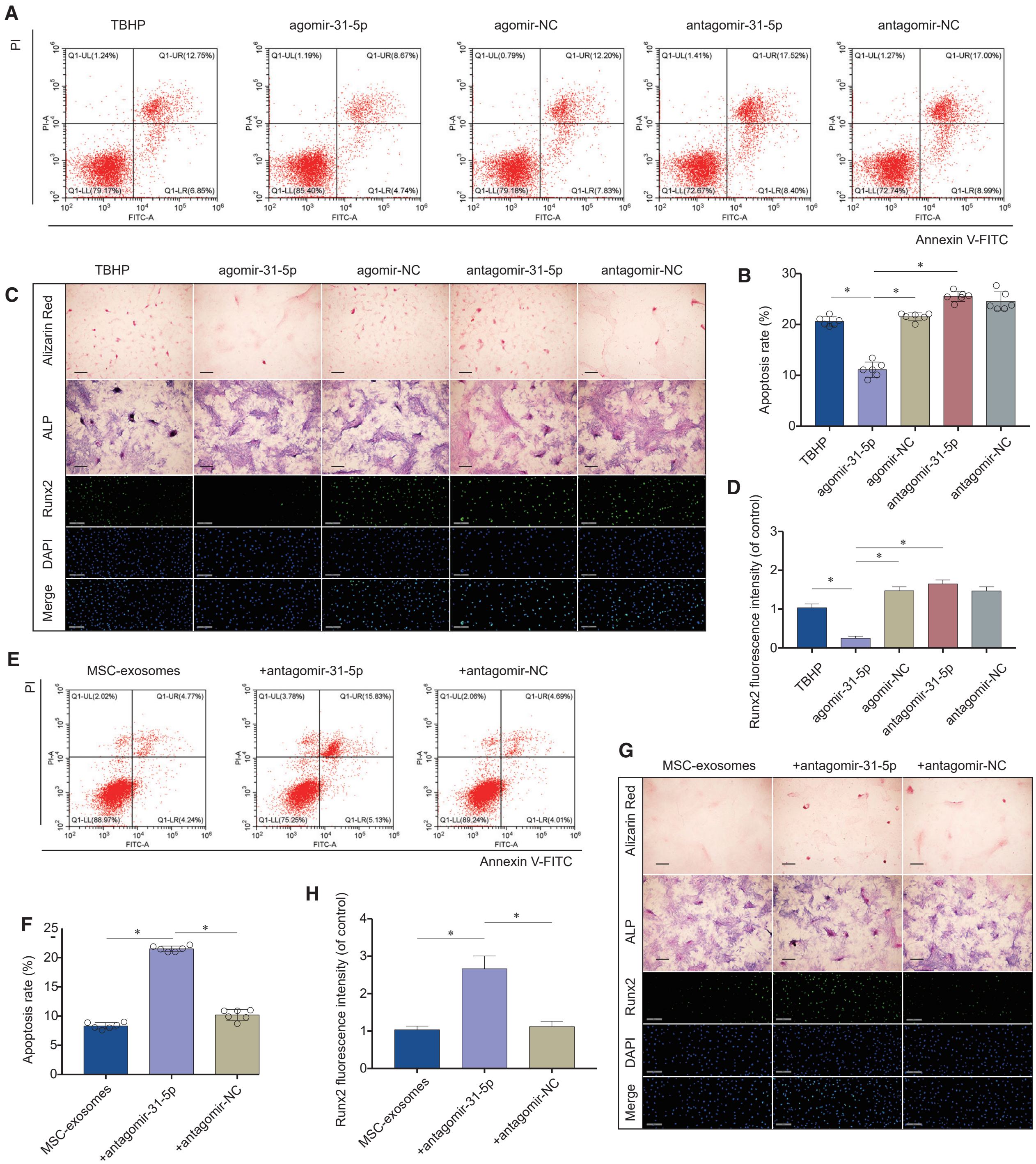

Figure 5. miR-31-5p in MSC-Exosomes Inhibited Apoptosis and Calcification in EPCs under Oxidative Stress

(A) Impact of miR-31-5p on EPC apoptosis was examined using the flow cytometry assay. (B) Agomir-31-5p significantly alleviated EPC apoptosis, whereas the antagomir-31-5p exacerbated apoptosis. (C) Impact of miR-31-5p on EPC calcification was evaluated using alizarin red staining, ALP staining (scale bars, 50 μm), and immunofluorescence staining of Runx2 (scale bars, 200 μm). (D) Agomir-31-5p significantly alleviated EPC calcification, whereas antagomir-31-5p exacerbated calcification. (E) Anti-apoptotic activities of miR-31-5p-depleted MSC-exosomes were evaluated using flow cytometry. (F) The percentage of the apoptotic cells was increased after silencing miR-31-5p in MSC-exosomes. (G) Anti-calcification activities of miR-31-5p-depleted MSC-exosomes were detected using alizarin red staining, ALP staining (scale bars, 50 μm), and immunofluorescence staining of Runx2 (scale bars, 200 μm). (H) The percentage of the Runx2-positive cell was increased after silencing miR-31-5p in MSC-exosomes. Data are mean ± SD. *p < 0.05, **p < 0.01, ***p < 0.001.

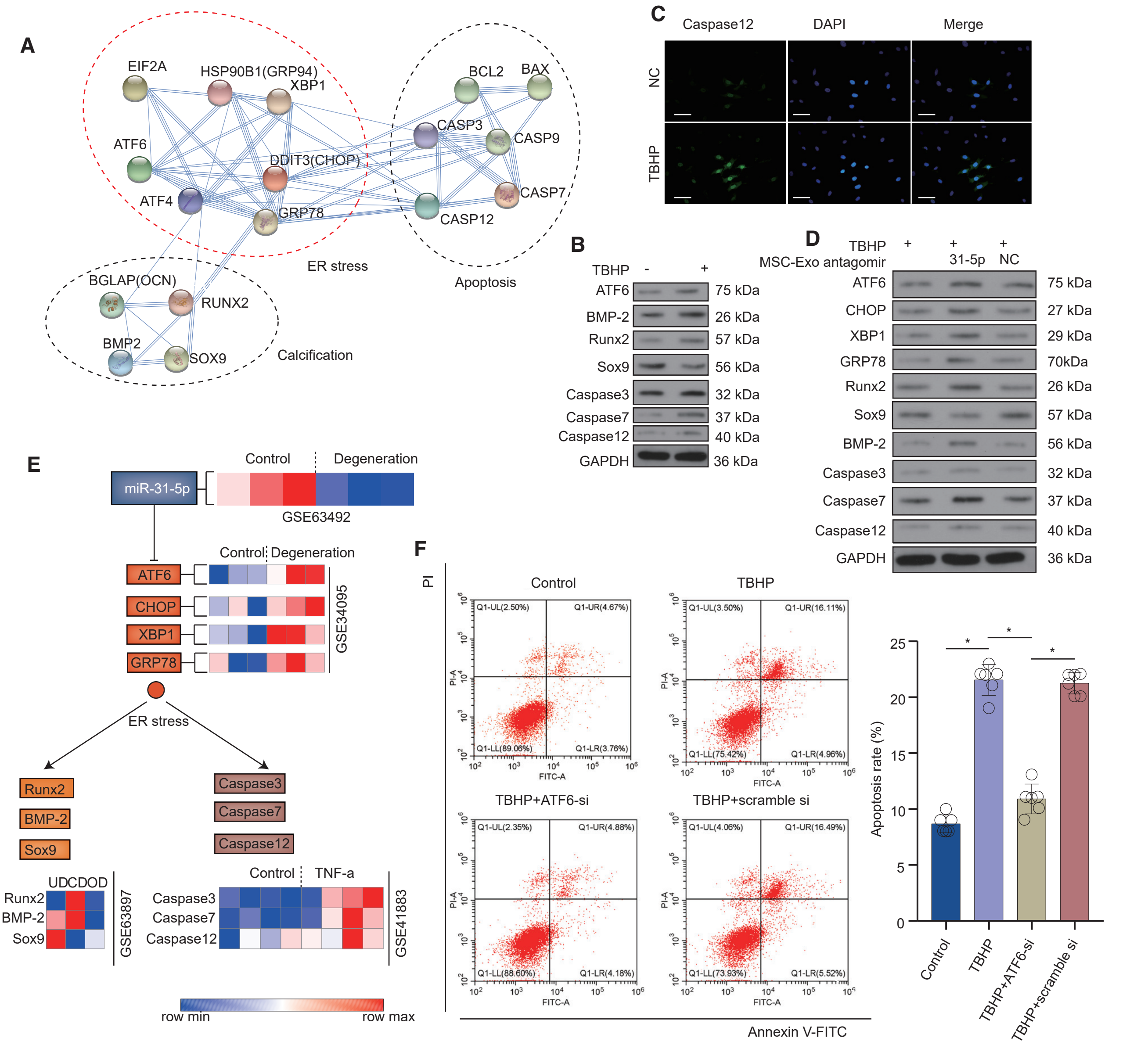

Figure 6. Inhibition of Apoptosis and Calcification via the ATF6-Related ER-Stress Pathway

(A) A graphic representation of the turquoise module using string (https://string-db.org/cgi/input.pl). (B) ATF6, apoptosis-related proteins, and calcification-related proteins in EPCs after TBHP treatment. (C) Immunofluorescence of caspase-12 protein in EPCs after TBHP treatment (scale bars, 100 μm). (D) miR-31-5p-deficient MSC-exosomes lost the ability of inhibition in ER-stress, apoptosis, and calcification. (E) Signal transduction pathways and expressions of related genes in the GEO database. (F) ATF6 siRNA significantly decreased apoptosis in EPCs induced by TBHP. Data are mean ± SD. *p < 0.05, **p < 0.01, ***p < 0.001.

structure was more intact in the MSC-exosome group than in the noninjection group, indicating that MSC-exosomes had beneficial effects for protecting CEP. The results also revealed that NP tissues were better preserved in the MSC-exosome group, implying that MSC-exosomes may also be beneficial for NP tissues (Figure 7D). Alcian blue (blue) staining results also showed similar effects (Figure 7E). Taken together, the histological score of the noninjection group was markedly higher compared with the MSC-exosome group (Figure 7F). The western blot results revealed that MSC-exosome injection had a positive effect on inhibiting apoptosis and calcification in CEP tissues from IVDD (Figure 7G). We additionally used the TUNEL 3,3′-diaminobenzidine (DAB) method to stain apoptotic cells in the CEP. Consequently, the percentage of DAB-positive cells in the MSC-exosome group was lower than in the noninjection group (Figures 7H and 7I). Immunohistochemical staining also showed that the percentage of Runx2-positive cells in the MSC-exosome group

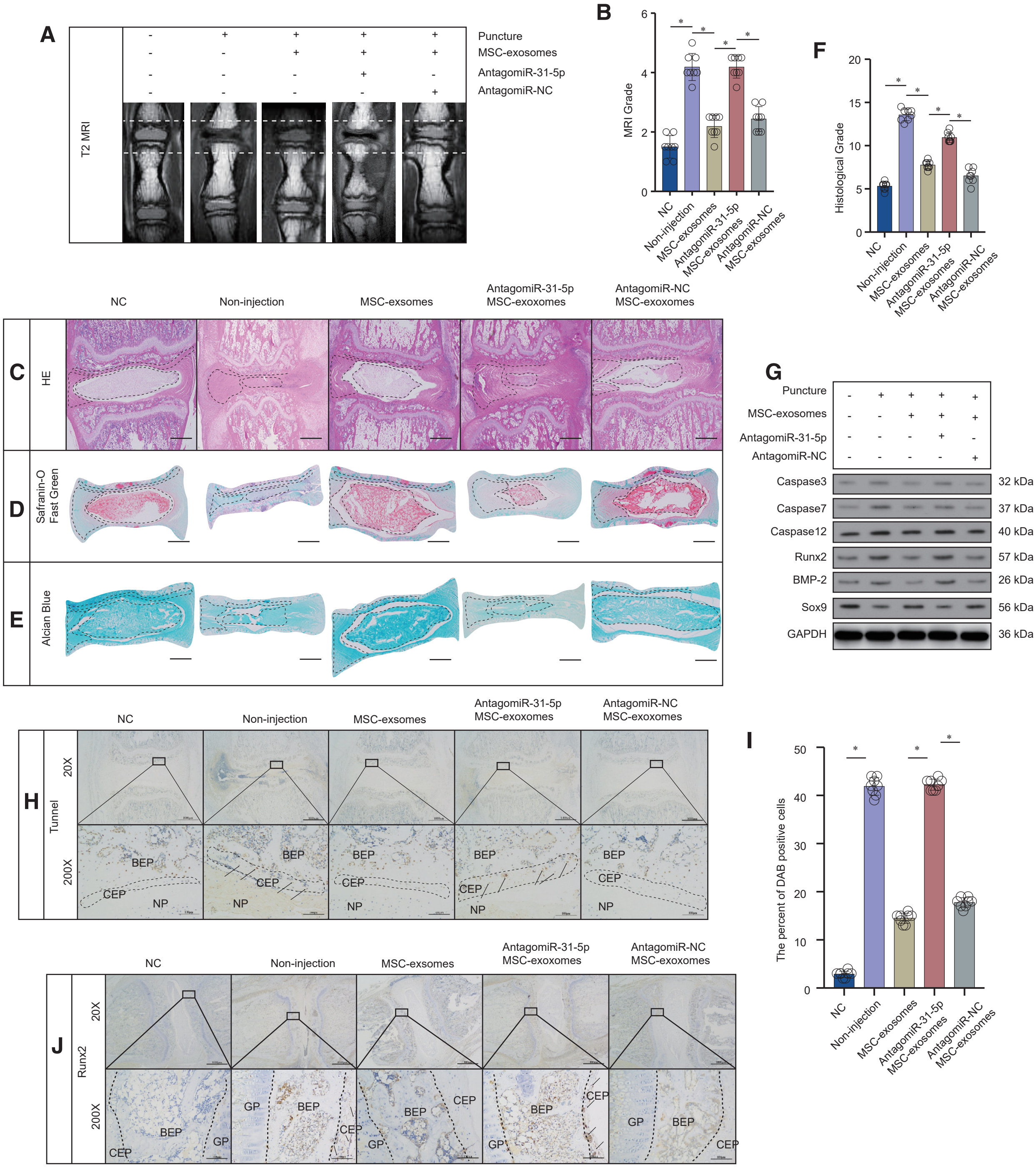

Figure 7. Sub-Endplate Injection of MSC-Exosomes Ameliorated IVDD in Rat Models

(A) MRI of the rat tails in different groups. (B) The scores of MRI in different groups. (C) H&E staining of rat-tail IVD sections in different groups (scale bars, 1,000 μm). (D) Safranin-O staining of rat-tail IVD sections in different groups (scale bars, 1,000 μm). (E) Alcian blue staining of rat-tail IVD sections in different groups (scale bars, 1,000 μm). (F) The histological grades of rat-tail IVD sections in different groups. (G) Apoptosis- and calcification-related proteins from CEP in different groups. (H) TUNEL staining (DAB) of rat-tail IVD sections, 20× (scale bars, 1,000 μm) and 200× (scale bars, 100 μm). Arrows indicate DAB-positive cells. (I) The percentage of DAB-positive cells in CEP in different groups. (J) The immunohistology staining of Runx2-positive cells in CEP in different groups, 20× (scale bar, 1,000 μm) and 200× (scale bars, 100 μm). Arrows indicate Runx2-positive cells. BEP, bony endplate; GP, growth plate. Data are mean ± SD. *$p < 0.05$, **$p < 0.01$, ***$p < 0.001$.

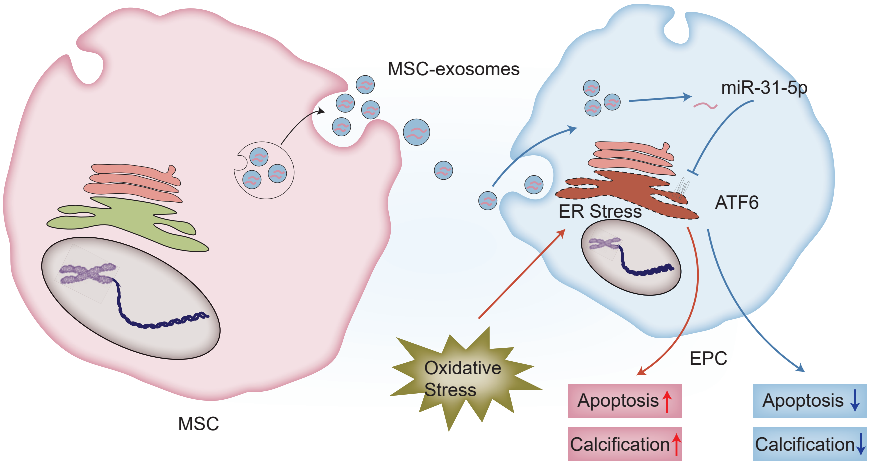

Figure 8. A Diagrammatic Sketch of Protective Effects of MSC-Exosomes in EPCs under Oxidative Stress

Oxidative stress leads to ER-stress-related apoptosis and calcification in EPCs. MSC-exosomes protect EPCs against apoptosis and calcification through a miR-31-5p/ATF6-related ER-stress pathway.

decreased compared to the noninjection group (Figure 7J), indicating that the MSC-exosome group reduced calcification in CEP compared to the noninjection group. However, after the miR-31-5p levels were downregulated in MSC-exosomes, these effects were significantly inhibited.

DISCUSSION

Although MSC-exosomes have advantageous effects on IVD in physiological and pathological conditions,[20,22] their effects and mechanisms of action on EPCs are minimally understood. Here, we established that in primary EPCs, MSC-exosomes inhibited apoptosis and calcification. We found that this mechanism was, at least partly, controlled by miR-31-5p, which can be transmitted into EPCs, thereby disrupting ATF6-ER-stress and regulating cell function.

Exosomes are extracellular vesicles (EVs) found in various cells types that can deliver miRNAs to recipient cells.[21] Although systemic delivery of exosomes is generally considered the easiest approach, biodistribution patterns indicate accumulation in the liver, spleen, and lungs.[23] Especially when taking the avascular nature of IVD into account, local delivery in the sub-endplate region was regarded as a good alternative choice. Meanwhile, sub-endplate injection can avoid repeated puncture in tail disc tissues. The perichondrium region adjacent to the epiphyseal plate and the outer zone of the AF has been suggested to be the IVD stem cell niche.[24,25] Sub-endplate injection also simulated bioinspired endogenous repair strategies.

In our results, MSC-exosomes reduced the apoptosis and calcification of EPCs, thereby inhibiting degeneration of CEP. Meanwhile, MSC-exosomes alleviated IVDD *in vivo*. Previous evidence *in vivo* and *in vitro* indicates that oxidation products are extensively presented in IVDD.[26,27] Previous research evidence indicated that oxidative stress products elevated the formation of cardiovascular cell calcification.[28,29] Recent studies indicated that oxidative stress also induced apoptosis and calcification in EPCs.[4,30,31] These studies implied that oxidative stress was a frequent pathological condition for apoptosis and calcification in cells, EPCs included.[32] Hence, we examined the mechanism of MSC-exosomes on EPCs apoptosis and calcification in the TBHP-induced oxidative stress system. In this study, we found that MSC-exosomes downregulated the expression of apoptosis-related proteins, suggesting that MSC-exosomes protected EPCs from apoptosis induced by oxidative stress. At the same time, MSC-exosomes also inhibited calcification induced by oxidative stress. Taken together, these results indicated that MSC-exosomes inhibited apoptosis as well as calcification in EPCs under oxidative stress.

As an important mediator of MSC-exosomes, miRNAs provide enduring therapeutic effects and basic changes in the local microenvironment.[33] In recent years, many studies have reported different miRNAs and their role in IVDD.[34,35] Three downmodulated miRNAs (miR-31-5p, miR-124a, and miR-127-5p) are frequently reported miRNAs in IVDD tissues.[18] In this study, we reported the high expression of miR-31-5p in MSC-exosomes and established that miRNA negatively regulates apoptosis and calcification. After insertion of miR-31-5p into EPCs via transfection, their apoptosis and calcification were significantly reduced. We incubated the EPCs with miR-31-5p-deficient MSC-exosomes. The suppressing impacts of MSC-exosomes on ER-stress-related apoptosis and calcification were inhibited, implying that MSC-exosomes inhibited the ER-stress-related apoptosis and calcification of EPCs via miR-31-5p.

In our study, we identified ATF6 as the target gene of miR-31-5p, thereby confirming the mechanism of miR-31-5p in mediating EPC apoptosis and calcification. When miR-31-5p was upregulated, ATF6 no longer promoted ER-stress in EPCs, resulting in reduced EPC apoptosis and calcification. Recently, studies reported that oxidative stress induced ER-stress in EPCs.[36] In addition, previous studies reported that ATF6 interacted with ER-stress elements to induce transcription factors to move into the nucleus, which in turn, led to the upregulation of genes related to unfolded proteins, such as CHOP, GRP78, and XBP1.[37] Herein, we found that MSC-exosomes downregulated the expression of ATF6, CHOP, XBP1, and GRP78, suggesting that MSC-exosomes had protective effects on oxidative stress-induced ER-stress in EPCs. Taken together, we provide insights into the beneficial effect of MSC-exosomes on EPCs. The MSC-exosome-mediated transfer of miR-31-5p had the advantageous effect on EPCs, possibly though ATF6-related ER-stress inhibition.

Previous studies also showed that the NP cells were preserved in the MSC-exosome-treated group compared with the untreated group *in vitro* studies.[20,22] Interestingly, aside from NP cells, the EPCs were also better preserved in the MSCs-exosome-treated group *in vitro* study. These results suggested that MSC-exosomes may contribute to IVDD therapeutics by targeting both NP and CEP. Our *in vivo* results also supported it.

In conclusion, our study indicated that MSC-exosomes prevented EPCs from apoptosis and calcification, at least partially, through miR-31-5p (Figure 8). Besides, miR-31-5p disrupted ATF6 and thus inhibited ER-stress-related apoptosis and calcification in EPCs under oxidative stress, and sub-injection of MSC-exosomes alleviates IVDD *in vivo*. Therefore, we provide a prospective therapeutic strategy for IVDD.

MATERIALS AND METHODS

Ethics Statement

All animal studies on the surgical intervention, treatment, and post-surgery animal care were approved by the Fudan University Animal Care and Use Committee.

Cell Isolation and Culture

The Sprague-Dawley rats (100–150 g) were sacrificed after anesthetization using isoflurane gas. We collected the CEP tissues using a dissecting microscope. The tissue was digested using 0.2% type II collagenase (Sigma-Aldrich) at 37°C for 4 h.[38] Next, we incubated the digested tissue as a monolayer in DMEM (Gibco, Invitrogen, Grand Island, NY, USA) containing antibiotics (1% penicillin/streptomycin) and 10% fetal bovine serum (FBS; HyClone, Logan, UT, USA) under the conditions of 5% CO_2 and 37°C. When the cells were 70%–80% confluent (appropriate density), 0.25% trypsin-EDTA (Gibco, Invitrogen) was used to extract the cells. Next, we transferred the EPCs to a 10-cm new culture plate at an appropriate density. We replaced the complete medium daily with fresh medium and used the EPCs of the previous two and three passages in the experiments.

Exosome Isolation

We isolated, cultivated, and characterized of MSCs from the vertebral body in anterior cervical corpectomy and fusion (ACCF) surgery. After 48 h, the culture supernatant was harvested. We harvested the culture supernatant via centrifugation at 3,000 × *g* for 10 min, then at 2,000 × *g* for 30 min, and then ultracentrifuged on an Optima L-100XP ultracentrifuge (Beckman Coulter, Brea, CA, USA) at 20,000 × *g* for 30 min. At every step, we transferred the supernatant into a clean tube and immediately resuspended the pellet in phosphate-buffered saline (PBS). The supernatant was filtered through a 0.22-μM filter, aliquoted, stored at −80°C, and then ultracentrifuged at 4°C for 2 h at 120,000 × *g*. The final volume was 200 μL. Subsequently, we washed the EV pellet in PBS at 120,000 × *g* for 2 h at 4°C and then resuspended in PBS. We used exosomes from normal human fibroblasts (Stem Cell Bank, Chinese Academy of Sciences, Shanghai) as the control.

Exosome Characterization and Uptake by EPCs

The exosome morphology was documented using TEM. The exosomes were confirmed based on the expression of signature markers (TSG101, CD9, and CD63) using western blot assays. We utilized the Nanosizer instrument (Malvern Instruments, Malvern, UK) in the DLS analyses. We incubated the purified MSC-exosomes with PKH26 (Sigma-Aldrich) for 5 min at room temperature. After washing twice with PBS via 120,000 × *g* centrifugation for 90 min, we resuspended the labeled exosomes in the basal medium and subsequently incubated them with EPCs at 37°C for 6 h. The uptake of labeled particles by EPCs was measured via immunofluorescence staining.

Alizarin Red Staining and ALP Staining

The EPCs were treated with TBHP (2 h), and then cells were cultured in routine DMEM medium (without TBHP) for 6 days. After that, the EPCs were washed three times with PBS and fixed with 4% paraformaldehyde for 15 min and with alizarin red solution (Beyotime, Shanghai, China) for 30 min at 37°C. The stained cells were observed, and images were captured with an inverted microscope (Nikon, Tokyo, Japan). A BCIP/NBT alkaline phosphatase color development kit (Beyotime, Shanghai, China) was utilized based upon provided directions. Briefly, cells were washed three times by using PBS and fixed with 4% paraformaldehyde for 15 min. BCIP/NBT substrate was then used to treat cells for 24 h, the stained cells were observed, and images were captured with an inverted microscope (Nikon, Tokyo, Japan).

Flow Cytometry

We utilized Annexin V-fluorescein isothiocyanate (FITC) and/or propidium iodide (PI) double-standard staining to examine cell apoptosis. We collected the cells 48 h following transfection and then adjusted the concentration to 1×10^6 cells/mL, followed by fixing, using 70% ice-cold ethanol solution at 4°C overnight. After that, we centrifuged a 100-μL cell suspension (no less than 10^6 cells/mL). We then resuspended the cells in 200 μL of the binding buffer, followed by subsequent mixing with 10 μL Annexin V-FITC and 5 μL PI for 15 min in the dark. After that, we added 300 μL of the binding buffer. Finally, fluorescence (excitation wavelength = 488 nm) was detected by flow cytometry.

miRNA Array and Data Analysis

We considered the miRNAs with more than 2-fold difference and statistical significance ($p < 0.05$) between groups as differentially expressed. Heatmap analyses were performed using MORPHEUS software (https://software.broadinstitute.org/morpheus/). Four databases were used to identify target genes via the Venn analysis.

qRT-PCR Analysis

We suspended the MSC-exosomes in PBS containing 5% Triton. The MSC-exosomes were added with 0.4 mg/mL RNase A and incubated at 37°C for 10 min. After that, we added 0.1 mg/mL proteinase K and incubated at 37°C for 20 min. The miR-31-5p levels were analyzed by qRT-PCR. The sequences of primer and probe utilized are itemized in Table S2.

Western Blot

We lysed tissue or cells on ice using 1% protease suppressor (AD1008; Aspen, South Africa) and lysis buffer (AS1004; Aspen, South Africa). We collected and separated the protein fractions via SDS-PAGE and then embedded onto the nitrocellulose membrane (IPVH00010; Millipore, USA). After that, we blocked the membrane using 5% skimmed milk, and then primary antibodies were conjugated at 4°C overnight and then detected using horseradish peroxidase (HRP)-conjugated secondary antibodies (AS1058; Aspen, South Africa). For protein visualization, we utilized the chemiluminescence detection system (LiDE110; Canon, Japan), per the procedure outlined by the manufacturer. The antibodies used in this study consisted of anti-Runx2 (1:1,000), anti-BMP-2 (1:500), anti-caspase-3 (1:500), anti-caspase-7 (1:500), anti-caspase-9 (1:500), anti-caspase-12 (1:500), anti-Sox9 (1:500), anti-ATF6 (1:500), anti-CHOP (1:1000), anti-XBP1 (1:500), anti-GRP78 (1:1,000), and anti-glyceraldehyde 3-phosphate dehydrogenase (GAPDH) (1:10,000), all bought from Abcam, USA.

Transfection

Briefly, agomir-31-5p, agomir-negative control (NC), antagomir-31-5p, and antagomir-NC (GenePharma, Shanghai) were transfected using Lipofectamine 3000 (L3000001; Thermo Fisher Scientific, USA) reagent at 200 μM, per the procedure of the manufacturer. Similarly, we used Lipofectamine 3000 in the transfection of cells with siRNA oligos. We transfected the cells with ATF6 siRNA (RiboBio, Guangzhou, China) at 50 nM.

Luciferase Reporter Assay

Amplification of the rat ATF6 3′ UTR region with the binding sequence of miR-31-5p from the mouse genomic DNA was accomplished via PCR. Subsequently, we subcloned the amplicons in the pGL3 vector (E1741; Promega, USA). The sequence was mutated using the QuikChange site-directed mutation kit (210518; Stratagene, USA). Transient transfection of the EPCs (2.5×10^5 cells per well) was performed using the Lipofectamine 3000 reagent (L3000001; Thermo Fisher Scientific, USA) in a 24-well plate. We transfected 100 ng luciferase and 10 rng Renilla luciferase (pRL-TK) plasmid (E2241; Promega, USA) Renilla luciferase plasmid into cells and then used the dual-luciferase report analysis system (E1910; Promega, USA), per the instructions outlined. Quantification was performed using a photometer (GloMax; Promega, USA), and the enzyme activities of firefly and Renilla luciferases standardized.

RNA FISH

We performed the RNA FISH assay in EPCs. Blue fluorescence (4′,6-diamidino-2-phenylindole [DAPI]) designated cell nucleus, whereas green fluorescence (Alexa 488) designated ATF6 mRNA, and red fluorescence (Cy-5) designated miR-31-5p. We utilized the BX53 microscope (Olympus, Tokyo, Japan) in acquiring the images. The sequences of primer and probe utilized are itemized in Table S2.

Immunofluorescence Staining

With the use of PBS, we washed the EPCs thrice. After that, they were fixed using 4% paraformaldehyde for 15 min and infiltrated using 0.5% Triton X-100 for 20 min. We then blocked the cells using 1% goat serum albumin for 1 h, followed by overnight incubation at 4°C with the primary antibodies (caspase-12, dilution 1:200; Runx2, dilution 1:200; ATF6, dilution 1:200; CHOP, dilution 1:200; KDEL, dilution 1:200; all from Abcam, USA). Subsequently, we incubated the cells with the secondary antibodies for 1 h. We then used the DAPI reagent to stain the nuclei for 5 min. Finally, the BX53 microscope (Olympus, Tokyo, Japan) was utilized to acquire the images of the sections or cells.

Sub-Endplate Injection of MSC-Exosomes in Rat Models of IVDD

We obtained 40 adult female Sprague-Dawley rats (200–250 g) from the Laboratory Animal Research Institute of Fudan University and kept them in a controlled environment with standard conditions, temperature, and 12 h light and dark cycles. We randomly divided the rats into 5 groups: control group, IVDD (no injection) group, MSC-exosome group, antagomir-31-5p MSC-exosome group, and the antagomir-NC MSC-exosome group. All of the rats were anesthetized using isoflurane gas. We established the model of IVDD, as previously described. We chose Co7–8 coccyx intervertebral space for the operation. With the use of the 18G needles, we punctured the tail discs of the rats, followed by retaining the needles in the discs for 1 min. The rats in the MSC-exosome group, antagomir-31-5p MSC-exosome group, and antagomir-NC MSC-exosome group were sub-endplate injected with MSC-exosome, antagomir-31-5p MSC-exosome, or antagomir-NC MSC-exosome, once a week, up to 9 weeks, when they were sacrificed.

MRI Examination

Following 9 weeks of surgery and injections, we anaesthetized all of the rats using isoflurane gas. We selected the sagittal T2-weighted images using a 7.0-T MR (MRBioSpec70/20USR). Three orthopedic researchers assessed the MRI images. We used a 5-scale grading system in the MRI grading, per the Pfirrmann grade.

Histological Evaluation

We collected the tails of rats from five groups. After that, fixation of the tissues in 10% neutral-buffered formalin for 1 week was conducted, followed by decalcification in EDTA for 21 days, and then they were embedded in paraffin. Subsequently, we cut the tissues into 5 μm sections. With the use of H&E, Alcian blue, and Safranin-O methods, we stained these sections.

Immunohistochemistry Staining

We incubated the sections with 3% hydrogen peroxide for 10 min. Then, we washed them thrice using PBS. Next, we incubated the sections in 0.1% trypsin for 20 min, followed by washing thrice using PBS. With the use of 1% goat serum albumin, we blocked the sections at 37°C for 1 h, and then the primary antibodies of Runx2 (1:800 dilution; Abcam, USA) conjugated via incubation. The control group was incubated with nonspecific immunoglobulin G (IgG). Subsequently, we washed them thrice with PBS, and then the HRP-conjugated secondary antibody conjugated via incubation at 37°C for 1 h. Finally,

the images of the sections were obtained using a BX53 microscope (Olympus, Tokyo, Japan).

In Situ Apoptosis Analysis

We used the *In Situ* Apoptosis Detection Kit (Abcam, USA) to evaluate apoptosis. First, the dewaxing of the paraffin sections in xylene was performed. Then, rehydration of the sections in graded alcohols was done, followed by incubation with proteinase K. After that, we added 3% H_2O_2 to degrade the endogenous peroxidase. With the use of the terminal deoxynucleotidyl transferase (TdT), we labeled the apoptotic cells. TdT catalyzed the addition of biotin-labeled deoxynucleotides and then incubated streptavidin- HRP conjugated via incubation. We treated the positive control group DNase I and the NC group with water instead of TdT. We used the DAB substrate to detect the signal.

Statistical Analysis

The data were designated as the mean ± SD. We utilized the unpaired two-tailed Student's t test to conduct statistical analysis between two groups. In the multiple group comparisons, we conducted one-way analysis of variance (ANOVAs). All data were analyzed using Prism version 8.0 (La Jolla, CA, USA) software or SPSS software version 21.0 (IBM, Armonk, NY, USA). For all analyses, $p < 0.05$ indicated statistical significance.

SUPPLEMENTAL INFORMATION

Supplemental Information can be found online at https://doi.org/10.1016/j.omtn.2020.09.026.

AUTHOR CONTRIBUTIONS

L.X., M.L., and J.J. designed the experiments. L.X., Z.C., W.H., H.W., F.Z., and X.M. performed the experiments and acquired the data. L.X., X.M., J.T., J.G., X.X., C.Z., and F.L. analyzed the data. L.X., C.Z., H.W., and J.J. supervised the project and wrote the manuscript.

CONFLICTS OF INTEREST

The authors declare no competing interests.

ACKNOWLEDGMENTS

This work was supported by the National Natural Science Foundation of China (81972109, 81802145, 81772388, and 81972093).

REFERENCES

1. Maniadakis, N., and Gray, A. (2000). The economic burden of back pain in the UK. Pain *84*, 95–103.
2. Luoma, K., Riihimäki, H., Luukkonen, R., Raininko, R., Viikari-Juntura, E., and Lamminen, A. (2000). Low back pain in relation to lumbar disc degeneration. Spine *25*, 487–492.
3. Zhu, Q., Gao, X., Levene, H.B., Brown, M.D., and Gu, W. (2016). Influences of Nutrition Supply and Pathways on the Degenerative Patterns in Human Intervertebral Disc. Spine *41*, 568–576.
4. Han, Y., Li, X., Yan, M., Yang, M., Wang, S., Pan, J., Li, L., and Tan, J. (2019). Oxidative damage induces apoptosis and promotes calcification in disc cartilage endplate cell through ROS/MAPK/NF-κB pathway: Implications for disc degeneration. Biochem. Biophys. Res. Commun. *516*, 1026–1032.
5. Sakai, D., and Andersson, G.B.J. (2015). Stem cell therapy for intervertebral disc regeneration: obstacles and solutions. Nat. Rev. Rheumatol. *11*, 243–256.
6. Liu, Y., Li, Y., Nan, L.P., Wang, F., Zhou, S.F., Feng, X.M., Liu, H., and Zhang, L. (2020). Insights of stem cell-based endogenous repair of intervertebral disc degeneration. World J. Stem Cells *12*, 266–276.
7. Huang, Y.C., Leung, V.Y.L., Lu, W.W., and Luk, K.D.K. (2013). The effects of microenvironment in mesenchymal stem cell-based regeneration of intervertebral disc. Spine J. *13*, 352–362.
8. Gruber, H.E., and Hanley, E.N., Jr. (1998). Analysis of aging and degeneration of the human intervertebral disc. Comparison of surgical specimens with normal controls. Spine *23*, 751–757.
9. Akyurekli, C., Le, Y., Richardson, R.B., Fergusson, D., Tay, J., and Allan, D.S. (2015). A systematic review of preclinical studies on the therapeutic potential of mesenchymal stromal cell-derived microvesicles. Stem Cell Rev. Rep. *11*, 150–160.
10. Chen, T.S., Lai, R.C., Lee, M.M., Choo, A.B.H., Lee, C.N., and Lim, S.K. (2010). Mesenchymal stem cell secretes microparticles enriched in pre-microRNAs. Nucleic Acids Res. *38*, 215–224.
11. Rani, S., Ryan, A.E., Griffin, M.D., and Ritter, T. (2015). Mesenchymal Stem Cell-derived Extracellular Vesicles: Toward Cell-free Therapeutic Applications. Mol. Ther. *23*, 812–823.
12. Latifkar, A., Hur, Y.H., Sanchez, J.C., Cerione, R.A., and Antonyak, M.A. (2019). New insights into extracellular vesicle biogenesis and function. J. Cell Sci. *132*, jcs222406.
13. Xiao, J., Pan, Y., Li, X.H., Yang, X.Y., Feng, Y.L., Tan, H.H., Jiang, L., Feng, J., and Yu, X.Y. (2016). Cardiac progenitor cell-derived exosomes prevent cardiomyocytes apoptosis through exosomal miR-21 by targeting PDCD4. Cell Death Dis. *7*, e2277.
14. Rupaimoole, R., and Slack, F.J. (2017). MicroRNA therapeutics: towards a new era for the management of cancer and other diseases. Nat. Rev. Drug Discov. *16*, 203–222.
15. Ameres, S.L., and Zamore, P.D. (2013). Diversifying microRNA sequence and function. Nat. Rev. Mol. Cell Biol. *14*, 475–488.
16. Ying, W., Riopel, M., Bandyopadhyay, G., Dong, Y., Birmingham, A., Seo, J.B., Ofrecio, J.M., Wollam, J., Hernandez-Carretero, A., Fu, W., et al. (2017). Adipose Tissue Macrophage-Derived Exosomal miRNAs Can Modulate In Vivo and In Vitro Insulin Sensitivity. Cell *171*, 372–384.e12.
17. Chen, Y., and Pfeifer, A. (2017). Brown Fat-Derived Exosomes: Small Vesicles with Big Impact. Cell Metab. *25*, 759–760.
18. Wang, X.Q., Tu, W.Z., Guo, J.B., Song, G., Zhang, J., Chen, C.C., and Chen, P.J. (2019). A Bioinformatic Analysis of MicroRNAs' Role in Human Intervertebral Disc Degeneration. Pain Med. *20*, 2459–2471.
19. Yu, T., Ma, P., Wu, D., Shu, Y., and Gao, W. (2018). Functions and mechanisms of microRNA-31 in human cancers. Biomed. Pharmacother. *108*, 1162–1169.
20. Liao, Z., Luo, R., Li, G., Song, Y., Zhan, S., Zhao, K., Hua, W., Zhang, Y., Wu, X., and Yang, C. (2019). Exosomes from mesenchymal stem cells modulate endoplasmic reticulum stress to protect against nucleus pulposus cell death and ameliorate intervertebral disc degeneration in vivo. Theranostics *9*, 4084–4100.
21. Lewis, J.M., Vyas, A.D., Qiu, Y., Messer, K.S., White, R., and Heller, M.J. (2018). Integrated Analysis of Exosomal Protein Biomarkers on Alternating Current Electrokinetic Chips Enables Rapid Detection of Pancreatic Cancer in Patient Blood. ACS Nano *12*, 3311–3320.
22. Cheng, X., Zhang, G., Zhang, L., Hu, Y., Zhang, K., Sun, X., Zhao, C., Li, H., Li, Y.M., and Zhao, J. (2018). Mesenchymal stem cells deliver exogenous miR-21 via exosomes to inhibit nucleus pulposus cell apoptosis and reduce intervertebral disc degeneration. J. Cell. Mol. Med. *22*, 261–276.
23. Piazza, N., Dehghani, M., Gaborski, T.R., and Wuertz-Kozak, K. (2020). Therapeutic Potential of Extracellular Vesicles in Degenerative Diseases of the Intervertebral Disc. Front. Bioeng. Biotechnol. *8*, 311.
24. Lyu, F.J., Cheung, K.M., Zheng, Z., Wang, H., Sakai, D., and Leung, V.Y. (2019). IVD progenitor cells: a new horizon for understanding disc homeostasis and repair. Nat. Rev. Rheumatol. *15*, 102–112.
25. Henriksson, H., Thornemo, M., Karlsson, C., Hägg, O., Junevik, K., Lindahl, A., and Brisby, H. (2009). Identification of cell proliferation zones, progenitor cells and a potential stem cell niche in the intervertebral disc region: a study in four species. Spine *34*, 2278–2287.

26. Su, T., Xiao, Y., Xiao, Y., Guo, Q., Li, C., Huang, Y., Deng, Q., Wen, J., Zhou, F., and Luo, X.H. (2019). Bone Marrow Mesenchymal Stem Cells-Derived Exosomal MiR-29b-3p Regulates Aging-Associated Insulin Resistance. ACS Nano *13*, 2450–2462.

27. Thomou, T., Mori, M.A., Dreyfuss, J.M., Konishi, M., Sakaguchi, M., Wolfrum, C., Rao, T.N., Winnay, J.N., Garcia-Martin, R., Grinspoon, S.K., et al. (2017). Adipose-derived circulating miRNAs regulate gene expression in other tissues. Nature *542*, 450–455.

28. Cheng, X., Ni, B., Zhang, F., Hu, Y., and Zhao, J. (2016). High Glucose-Induced Oxidative Stress Mediates Apoptosis and Extracellular Matrix Metabolic Imbalances Possibly via p38 MAPK Activation in Rat Nucleus Pulposus Cells. J. Diabetes Res. *2016*, 3765173.

29. Aghagolzadeh, P., Radpour, R., Bachtler, M., van Goor, H., Smith, E.R., Lister, A., Odermatt, A., Feelisch, M., and Pasch, A. (2017). Hydrogen sulfide attenuates calcification of vascular smooth muscle cells via KEAP1/NRF2/NQO1 activation. Atherosclerosis *265*, 78–86.

30. Zhang, Z., Lin, J., Tian, N., Wu, Y., Zhou, Y., Wang, C., Wang, Q., Jin, H., Chen, T., Nisar, M., et al. (2019). Melatonin protects vertebral endplate chondrocytes against apoptosis and calcification via the Sirt1-autophagy pathway. J. Cell. Mol. Med. *23*, 177–193.

31. Yuan, F.L., Xu, R.S., Ye, J.X., Zhao, M.D., Ren, L.J., and Li, X. (2019). Apoptotic bodies from endplate chondrocytes enhance the oxidative stress-induced mineralization by regulating PPi metabolism. J. Cell. Mol. Med. *23*, 3665–3675.

32. Ageta, H., and Tsuchida, K. (2019). Post-translational modification and protein sorting to small extracellular vesicles including exosomes by ubiquitin and UBLs. Cell. Mol. Life Sci. *76*, 4829–4848.

33. Zheng, Q., Li, X.X., Xiao, L., Shao, S., Jiang, H., Zhang, X.L., Sun, L.Y., and Xu, H.G. (2019). MicroRNA-365 functions as a mechanosensitive microRNA to inhibit end plate chondrocyte degeneration by targeting histone deacetylase 4. Bone *128*, 115052.

34. Chen, Z., Liu, M., Zhang, W., Deng, M., Zhou, Y., and Li, Y. (2020). miR-24-3p induces human intervertebral disc degeneration by targeting insulin-like growth factor binding protein 5 and the ERK signaling pathway. Life Sci. *243*, 117288.

35. Ji, M.L., Jiang, H., Zhang, X.J., Shi, P.L., Li, C., Wu, H., Wu, X.T., Wang, Y.T., Wang, C., and Lu, J. (2018). Preclinical development of a microRNA-based therapy for intervertebral disc degeneration. Nat. Commun. *9*, 5051.

36. Zhao, C.Q., Zhang, Y.H., Jiang, S.D., Jiang, L.S., and Dai, L.Y. (2010). Both endoplasmic reticulum and mitochondria are involved in disc cell apoptosis and intervertebral disc degeneration in rats. Age (Dordr.) *32*, 161–177.

37. Tao, Y.K., Shi, J., Yu, P.L., and Zhang, G.Q. (2018). The Role of Endoplasmic Reticulum Stress-related Apoptosis in Vascular Endothelium Pathogenesis. Biomed. Environ. Sci. *31*, 555–559.

38. Chen, D., Xia, D., Pan, Z., Xu, D., Zhou, Y., Wu, Y., Cai, N., Tang, Q., Wang, C., Yan, M., et al. (2016). Metformin protects against apoptosis and senescence in nucleus pulposus cells and ameliorates disc degeneration in vivo. Cell Death Dis. *7*, e2441.

OMTN, Volume *22*

Supplemental Information

MSC-Derived Exosomes Protect Vertebral Endplate Chondrocytes against Apoptosis and Calcification via the miR-31-5p/ATF6 Axis

Lin Xie, Zhenhao Chen, Ming Liu, Weibo Huang, Fei Zou, Xiaosheng Ma, Jie Tao, Jingkang Guo, Xinlei Xia, Feizhou Lyu, Hongli Wang, Chaojun Zheng, and Jianyuan Jiang

Figure S1

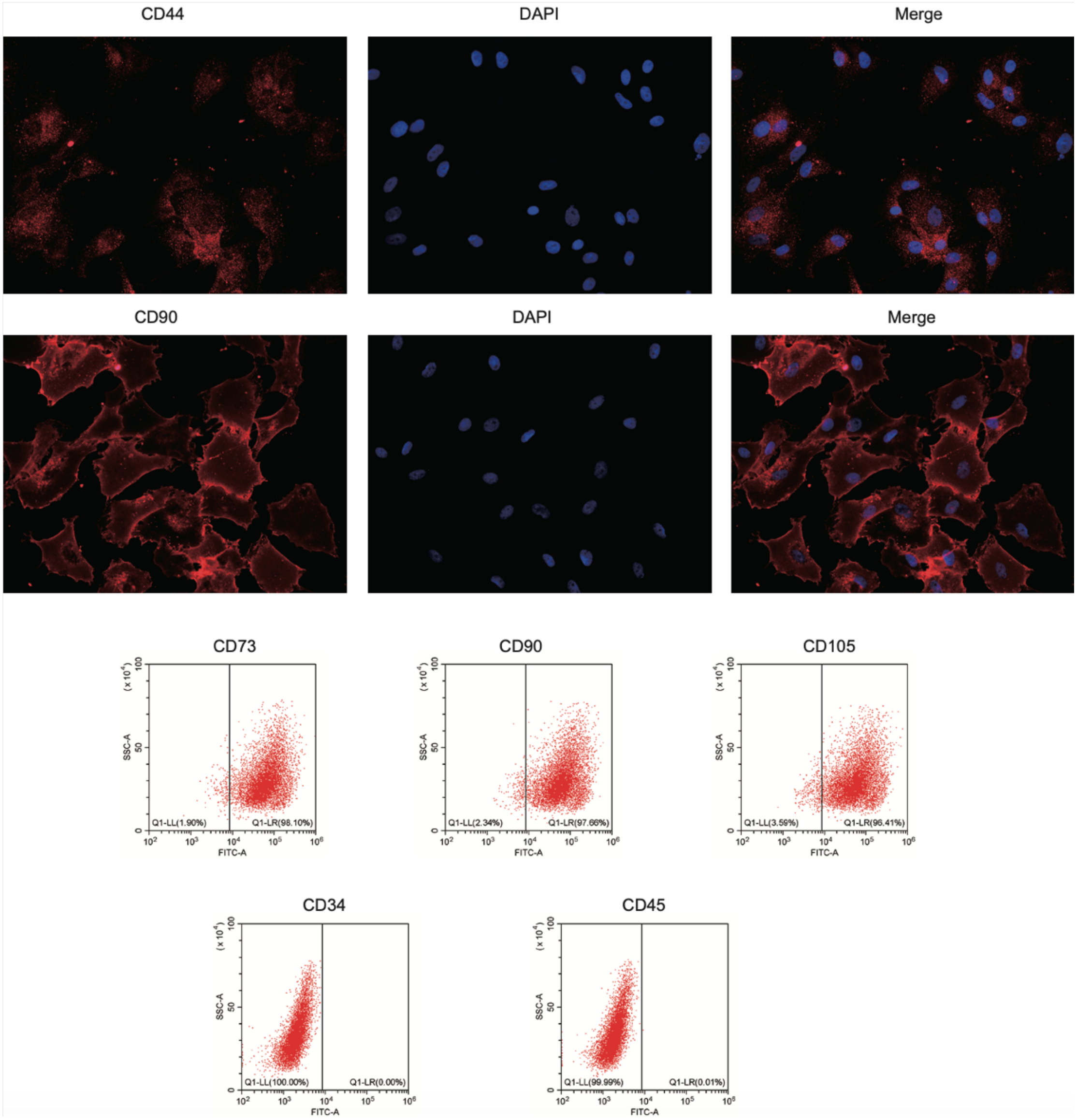

Figure S1. Analysis of expression patterns of CD44, CD105, CD73, and CD90, and deficiency of CD45 and CD34 surface molecules on MSCs using Immunofluorescence or flow cytometry.

Figure S2

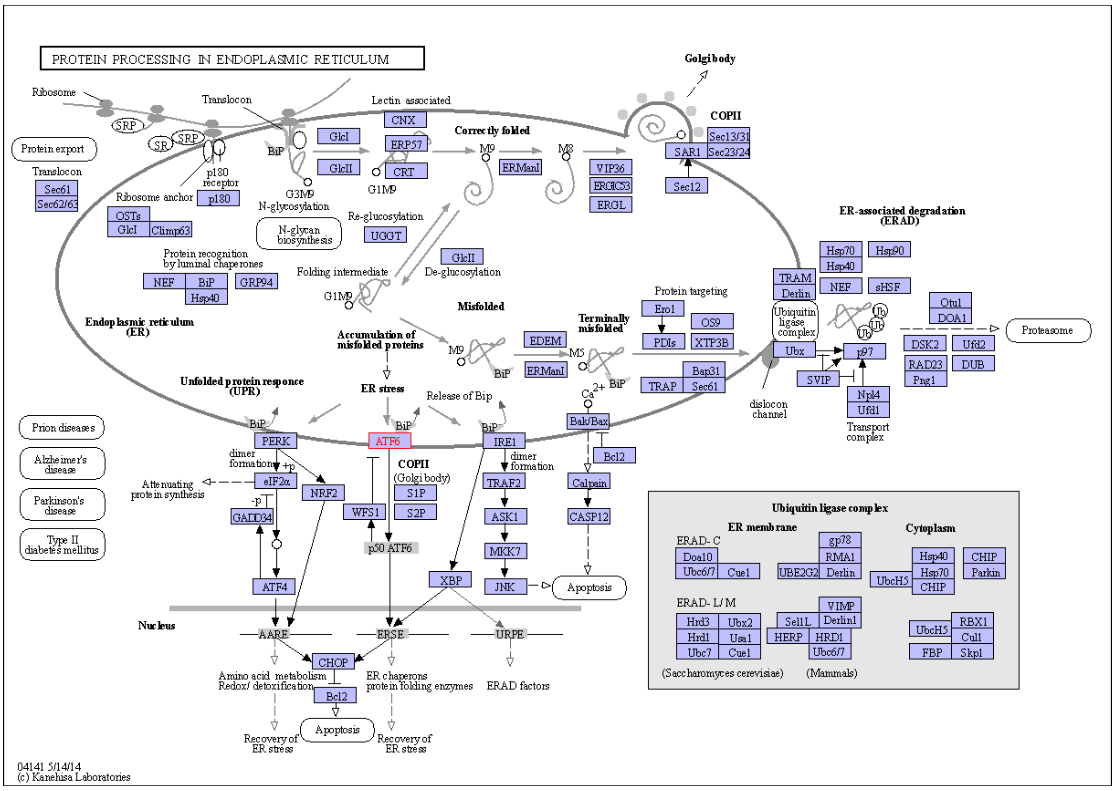

Figure S2. KEGG pathway showed that the direct effect of ATF6 was ER stress activation and related apoptosis pathway.

Table S1. MSCs and Endplate donors used for this study.

Donor ID	Sex	Age	Surgery Site	Site of vertebral body for MSCs	Site of intervertebral disc for EPCs
#1	Female	19	C4/5, C5/6		C5/6
#2	Male	27	C4/5, C5/6, C6/7		C5/6
#3	Male	25	C4/5, C5/6, C6/7		C5/6
#4	Male	28	C4/5, C5/6		C4/5
#5	Male	20	C4/5, C5/6, C6/7		C4/5
#6	Male	23	C4/5, C5/6		C4/5
#7	Male	21	C4/5, C5/6		C4/5
#8	Male	21	C4/5, C5/6, C6/7		C4/5
#9	Female	45	C4/5, C5, C5/6	C5	C4/5
#10	Female	53	C4/5, C5,	C5	C5/6

			C5/6		
#11	Male	49	C3/4, C4, C4/5	C4	C4/5
#12	Female	48	C4/5, C5/6		C4/5
#13	Male	49	C4/5, C5, C5/6	C5	C4/5
#14	Male	47	C3/4, C4, C4/5	C4	C4/5
#15	Female	47	C4/5, C5/6		C4/5
#16	Female	59	C4/5, C5/6		C4/5

MSCs, mesenchymal stem cells; EPCs, Endplate Chondrocytes; C, cervical vertebral body;

Table S2. Sequence used in this study.

	Sequences
U6 Forward	5'-CTCGCTTCGGCAGCACA-3'
U6 Reversed	5'-ACGCTTCACGAATTTGCGT-3'
miR-31-5p Forward	5'-CGGCGGAGGCAAGATGCTGGCA-3'
miR-31-5p Reversed	5'-CAACTGGTGTCGTGGAGTCGG-3'
agomiR-31-5p	AGGCAAGAUGCUGGCAUAGCU
agomiR-NC	UUUGUACUACACAAAAGUACU
antagomiR-31-5p	CAGCUAUGCCAGCAUCUUGCCU
Antagomir-NC	AAACAUGAUGUGUUUUCAUGAC
ATF6-siRNA Sense	AGTCGCCTTTTAGTCCGGTTC
ATF6-siRNA Antisense	CTGACTCCCAAGGCATCAAAT
Scramble siRNA Sense	ACCACAGTCCATGCCATCAC
Scramble siRNA Antisense	TCCACCACCCTGTTGCTGTA
